# Multiphysics finite element analysis of vertebral stability in simulated osteolytic lumbar defects treated with ^125^I brachytherapy and vertebroplasty

**DOI:** 10.3389/fbioe.2026.1764181

**Published:** 2026-07-23

**Authors:** Lulu Du, Jinwei Zhang, Jinhong Zhou, Yanbo Hu, Qiyu Sun, Jiaqi Yin, Chen Zhong, Min Li

**Affiliations:** 1 Department of Medical Oncology, The Postgraduate Training Base of Jinzhou Medical University, The 960th Hospital of People’s Liberation Army (PLA), Jinan, China; 2 Department of Radiology, Beijing Renhe Hospital, Beijing, China; 3 The Postgraduate Training Base of Jinzhou Medical University (The 960th Hospital of PLA), Jinan, Shandong, China; 4 The Postgraduate Training Base of Jinzhou Medical University (The 960th Hospital of PLA), Jinan, China; 5 The Postgraduate Training Base of Jinzhou Medical University, Jinzhou, China; 6 Department of Medical Oncology, The 960th Hospital of the PLA Joint Logistic Support Force, Jinan, Shandong, China; 7 Department of Nuclear Medicine, The 960th Hospital of the PLA Joint Logistic Support Force, Jinan, Shandong, China

**Keywords:** ^125^I seed brachytherapy, finite element analysis, metastatic bone destruction, percutaneous vertebroplasty (PVP), simulated osteolytic defects, vertebral mechanical stability

## Abstract

**Objective:**

This study evaluates the impact of radiation dose, thermal effects, and mechanical factors on vertebral stability and safety in a finite element model of simulated osteolytic defects and compared ^125^I seed brachytherapy alone with ^125^I brachytherapy combined with percutaneous vertebroplasty (PVP).

**Methods:**

A 3D FE model of the L3–L5 lumbar spine was reconstructed from CT data of a healthy adult subject. Idealized osteolytic defects were introduced into the anterior–middle L4 region at three volume grades: T ≤ 30%, 30% < T < 50%, and T ≥ 50%. Three scenarios were simulated for each grade: (i) osteolytic defect model, (ii) ^125^I seed implantation alone, and (iii) ^125^I-PVP combination. Ninety-day cumulative dose distributions (TG-43U1) were used to model dose-dependent degradation of material properties. PMMA polymerization was modeled using Arrhenius kinetics, and the temperature field was coupled into the structural analysis. Under physiological loading (500N + 7.5 N m), maximum displacement, maximum von Mises stress, peak temperature, and thermally damaged tissue volume fraction were calculated.

**Results:**

For T ≤ 30%, ^125^I implantation alone caused minimal changes in displacement and stress. Adding PMMA increased flexion displacement to 7.12 mm and maximum flexion von Mises stress to 36.29 MPa. For T > 30%, ^125^I implantation alone did not substantially improve mechanical performance. Compared with ^125^I implantation alone, ^125^I seed brachytherapy combined with PVP reduced flexion displacement from 8.20 to 4.36 mm and maximum flexion von Mises stress from 32.41 to 28.92 MPa in the 30% < T < 50% group. In the T ≥ 50% group, the combined treatment reduced flexion displacement from 8.81 to 4.76 mm and maximum flexion von Mises stress from 53.53 to 29.97 MPa. PMMA polymerization generated peak interface temperatures of 74 °C–84 °C and thermally damaged tissue fractions of 8%–25%, while spinal canal temperatures remained below 43 °C.

**Conclusion:**

For T ≤ 30%, ^125^I seed implantation maintained vertebral stability, whereas adding PVP increased local stiffness without clear mechanical benefits. For T > 30%, particularly T ≥ 50%, ^125^I seed brachytherapy combined with PVP improved stress distribution and displacement control compared with ^125^I alone. These findings represent model-based trends and should not be interpreted as failure-load predictions or clinical thresholds.

## 1 Introduction

The spine is one of the most common sites for bone metastasis of malignant tumors, with osteolytic destruction of the vertebral body representing the most common presentation (Hawkins et al., 2023). As tumor burden increases and bone mass progressively declines, patients often develop refractory pain, vertebral collapse, and spinal instability; in severe cases, compression of the spinal cord or nerve roots may occur. These complications markedly reduce quality of life. Treatment strategies therefore focus on pain relief, restoration or maintenance of spinal stability, and control of local tumor progression (Hawkins et al., 2023). Among minimally invasive interventions, percutaneous vertebroplasty (PVP) and ^125^I brachytherapy have demonstrated considerable clinical value (Yan et al., 2022; He et al., 2025).

^125^I seed implantation brachytherapy has been widely used for local control and palliative treatment of a variety of solid tumors, owing to its concentrated dose distribution, limited injury to surrounding normal tissues, and the feasibility of repeated procedures (Wu et al., 2023). Implanting ^125^I seeds into osteolytic vertebral lesions enables continuous low-dose-rate radiation delivery within the lesion area (Jiang et al., 2025), which inhibits tumor cell proliferation, alleviates pain, and provides patients with durable local control effects (Li et al., 2023). However, the spatial characteristics of the dose field are affected by multiple factors, including the complex anatomical structure of the vertebral body, the degree of bone destruction, and the spatial distribution of seeds (Qiang et al., 2024). Its long-term impacts on surrounding neural structures and normal bone tissues remain incompletely clarified.

Percutaneous vertebroplasty (PVP) involves the percutaneous injection of bone cement into a pathological vertebral body, providing rapid partial restoration of vertebral strength and improvement of the local mechanical environment (Li et al., 2023). This intervention can relieve pain and reduce the risk of further vertebral collapse. For vertebrae with osteolytic destruction, PVP is frequently combined with intralesional ablation or radiotherapy, with the goal of enhancing local tumor control while achieving mechanical stability (Sun et al., 2025). However, the polymerization of bone cement generates heat, and the consequent temperature elevation may affect both tumor cells and surrounding normal tissues (Hossain et al., 2025). Meanwhile, the filling morphology and volume fraction of bone cement alter the intravertebral stress distribution, potentially inducing new mechanical risks such as adjacent vertebral fractures. Additionally, the spatial interaction between bone cement and ^125^I seeds may modify the local radiation dose distribution and energy deposition characteristics. These intertwined factors result in a lack of systematic quantitative evidence regarding the comprehensive safety and optimization strategies of the combined minimally invasive regimen (^125^I seed brachytherapy combined with PVP) with respect to radiation, thermal effects, and mechanical responses.

Finite element analysis (FEA), a well-established numerical computation method, enables quantitative simulation of biological tissue responses under diverse physical fields based on anatomically realistic three-dimensional geometry and material properties (Wang and Wu, 2023). In recent years, FEA has been extensively used to investigate spinal biomechanics, stress redistribution after bone cement augmentation, and alterations in dose and temperature distributions under radiotherapy. However, studies on radiation-thermo-mechanical multiphysics coupled finite element models—incorporating ^125^I seed radiation, bone cement polymerization thermal effects, and the overall mechanical behavior of the vertebra–bone cement–surrounding soft tissue complex in the context of vertebral osteolytic destruction—remain relatively scarce. Most existing studies focus solely on a single physical field or simple dual-field coupling, failing to fully capture complex clinical treatment scenarios.

In spinal biomechanical research, FEA has been widely utilized to quantify the impact of osteolytic defect volume on vertebral stability, laying a foundation for elucidating the underlying instability mechanisms (Soltani et al., 2024). Recent CT- and micro-CT-based studies combined with digital volume correlation (DVC) have further advanced the experimental and computational assessment of metastatic vertebral mechanics (Palanca et al., 2016; Cavazzoni et al., 2023; Garavelli et al., 2025). DVC enables full-field internal displacement and strain measurements in vertebral bodies and has been shown to provide reliable strain estimates in natural and augmented vertebrae (Palanca et al., 2016). Subsequent studies demonstrated that metastatic bone microstructure does not preclude DVC-based strain assessment and that lesion type, size, and location strongly influence internal deformation patterns and failure localization in metastatic vertebrae (Cavazzoni et al., 2023; Gibson et al., 2025). More recently, DVC measurements have been used to validate CT-based subject-specific finite element models of human metastatic vertebrae, providing an important framework for linking image-derived lesion morphology to mechanical behavior (Garavelli et al., 2025). However, these studies primarily focused on experimental characterization and validation of metastatic vertebral mechanics, whereas the coupled effects of ^125^I dose deposition, PMMA polymerization heat, and post-treatment mechanical stability remain insufficiently investigated. Together with this CT/DVC evidence, multiple experimental and FEA studies have indicated that osteolytic defect volume acts as a primary factor governing vertebral mechanical responses and instability risk. Early three-dimensional poroelastic FEA demonstrated that under multi-parameter conditions (e.g., loading, bone mineral density, pedicle involvement, intervertebral disc degeneration), osteolytic defect volume exerts the most prominent influence on vertebral bulging (VB) and vertebral height loss (VH), with its contribution outweighing that of bone mineral density and loading rate (Whyne et al., 2003). Another study demonstrated that when the osteolytic defect volume accounts for approximately one-third of the vertebral body (≈33%), even under physiological loading (<750 N), a significant increase in local tensile strain and adjacent vertebral load transfer is observable—indicating that the structure has entered a phase of nonlinear stress response (Alkalay and Harrigan, 2016). *In vitro* experiments based on digital image correlation (DIC) have provided further direct evidence: when the anterior wall defect accounts for approximately 30% of the vertebral volume, the surface strain distribution transitions from uniform to concentrated, with the strain peak emerging in the defect region and acting as the actual initiation site of structural failure (Palanca et al., 2018). Giuseppe Salvatore et al. performed finite element analysis on the L3–L5 segment and found that a metastatic lesion occupying 15% of the vertebral volume induced only limited deformation under normal bone mineral density. In contrast, when the lesion volume reached 30%, vertebral bulging (VB) and vertebral height loss (VH) increased by 88% and 109%, respectively, relative to the intact vertebra (Palanca et al., 2018). Based on the aforementioned evidence and mechanical thresholds, this study categorized osteolytic defect volume (T) into three grades: T ≤ 30% (relatively stable grade), characterized by a small defect and preserved overall vertebral mechanical integrity; 30% < T < 50% (grade with significantly elevated mechanical risk), where stress concentration and strain acceleration zones arise; and T ≥ 50% (high-risk grade), in which the risk of structural instability is substantial and enhanced intervention may be warranted.

Prior experimental and finite element studies indicate that lesion volume may be a key determinant of vertebral stability, with involvement near one-third of the vertebral body associated with a shift toward accelerated mechanical deterioration (Palanca et al., 2018; Alkalay and Harrigan, 2016). However, this quantitative evidence is not easily translated into bedside decision-making. Clinical assessment still commonly relies on composite qualitative or semi-quantitative tools such as the Spinal Instability Neoplastic Score (SINS), which integrate pain, alignment, collapse, and posterior element involvement but do not represent anterior–middle vertebral body destruction as a continuous volumetric variable (Fisher et al., 2010). Consequently, SINS offers limited guidance on the tolerable extent of osteolytic loss under physiological loading and on when reinforcement (e.g., percutaneous vertebroplasty, PVP) is likely to be biomechanically beneficial for a given lesion geometry, particularly when radiation effects and cement polymerization may influence local mechanics. Specifically, there is no quantitative mapping from CT-derived osteolytic volume fraction to objective mechanical endpoints that could operationalize augmentation decisions (Costa et al., 2019). Importantly, the present FEA results do not replace clinical judgment. Instead, CT-derived osteolytic volume fraction may be used as an imaging-based mechanical descriptor to complement SINS (and neurological/oncologic factors) during multidisciplinary discussions, particularly in borderline or intermediate-instability cases (Soltani et al., 2024). To address this gap, we established a sequentially coupled dose–thermal–mechanical finite element framework linking volume-based stratification to objective stability metrics (displacement and von Mises stress), while incorporating dose-related material degradation and PMMA polymerization heat. We hypothesized that (H1) for limited defect volume (T ≤ 30%), ^125^I implantation maintains stability and routine PVP provides little additional mechanical benefit and may increase local stress due to stiffness mismatch (H2) for moderate-to-large defect volume (T > 30%), adding PVP reduces displacement and stress relative to ^125^I alone; and (H3) PMMA polymerization elevates interface temperature but keeps spinal canal temperatures below commonly cited neural injury thresholds, with thermal risk mainly localized to peri-cement trabecular bone.

## 2 Materials and methods

**FIGURE 1:**
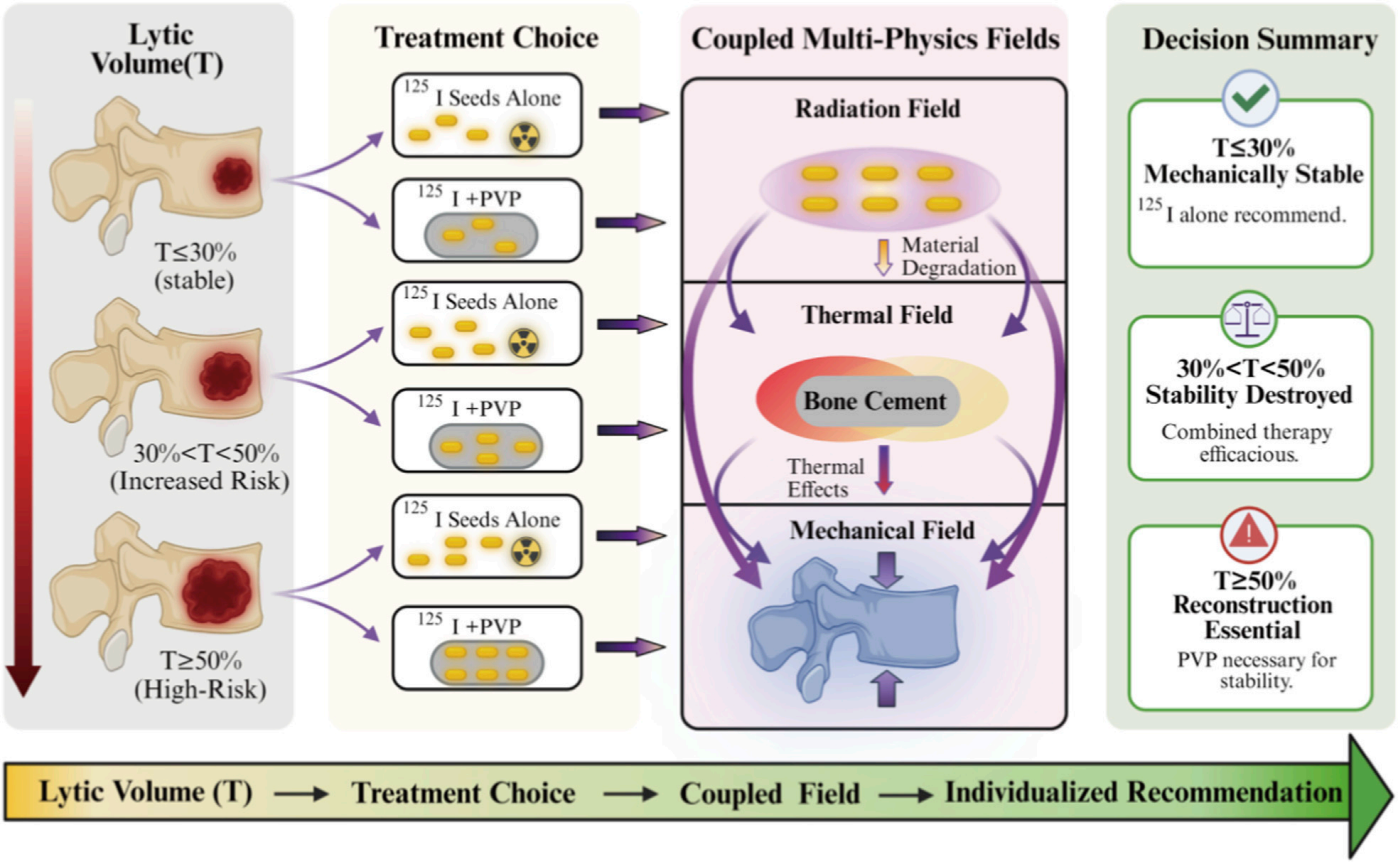
Overall workflow diagram.

The overall workflow of this study is illustrated in Figure 1.

### 2.1 Construction of the basic finite element model

**FIGURE 2:**
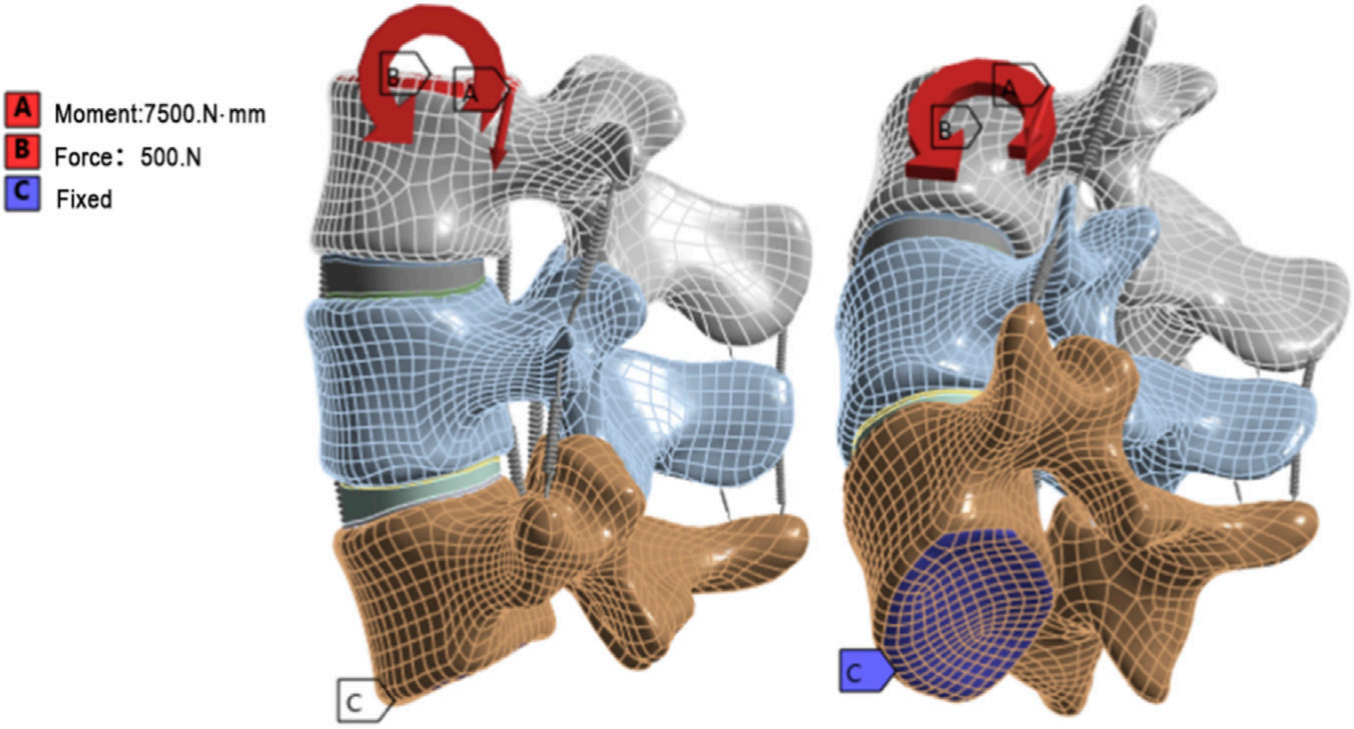
Mechanical model of spinal loading.

A healthy adult male (height: 178 cm; weight: 70 kg; no history of spinal disorders) was selected as the modeling subject. Spiral computed tomography (CT) scans of the L3–L5 segments were acquired using a 64-slice scanner with the following parameters: slice thickness of 0.625 mm and matrix size of 512 × 512. Digital Imaging and Communications in Medicine (DICOM) data were imported into Mimics 21.0 (Materialise, Belgium) for threshold-based segmentation and three-dimensional (3D) reconstruction (Widmer Soyka et al., 2016). Following surface optimization via Geomagic 2021 (3D Systems, United States), the geometric model was imported into SOLIDWORKS 2022 (Dassault Systèmes, France) to construct the structures of cortical bone, cancellous bone, endplates, intervertebral discs, and facet joints. The cortical bone shell and endplates were each set to a thickness of 1 mm, while the articular cartilage was set to 0.3 mm in thickness. The nucleus pulposus was defined as occupying 30%–40% of the intervertebral disc volume. After confirming “no interference” through assembly interference inspection, the model was imported into ANSYS (ANSYS Inc., United States) for finite element meshing (Pei et al., 2022; Bereczki et al., 2021; Zhang et al., 2022). SOLID187 elements were employed to represent solid structures, while ligamentous structures were simulated via spring elements. Material parameters were determined based on prior literature (Table 1). CT images were used only for geometric reconstruction and segmentation, not for density-based material calibration. No phantom-based or phantomless calibration was performed; therefore, each tissue/material type was assigned homogeneous, isotropic, linear elastic properties according to literature values. All degrees of freedom of nodes on the inferior surface of the L5 vertebra were constrained. A compressive preload of 500 N was applied to the superior surface of L3 to approximate the upper-body load transmitted through the lumbar spine under upright physiological conditions, as commonly used in lumbar finite element models (Rohlmann et al., 2009a; Chen H. et al., 2024). A pure moment of 7.5 N m was then applied to the same surface to simulate flexion, extension, left/right lateral bending, and left/right axial rotation. This moment magnitude has been widely used in lumbar spine biomechanical studies to reproduce physiological ranges of motion and facilitate comparison with previous experimental and finite element models (Panjabi et al., 1994; Rohlmann et al., 2009b; Chen H. et al., 2024) (Figure 2). Because the baseline geometry was reconstructed from a healthy subject rather than patient-specific metastatic vertebrae, the artificially introduced L4 lytic regions are referred to throughout as simulated osteolytic defects. The defect region was modeled as homogeneous, isotropic soft tissue with a Young’s modulus of 1 MPa to represent a compliant (Galbusera et al., 2018), weakly structured tissue and to reduce numerical instability associated with a zero-stiffness void. In the representative T ≥ 50% ^125^I seed brachytherapy combined with PVP model under flexion loading, a sensitivity analysis was performed by varying the modulus of the defect-filling tissue from 1 to 10 MPa (1–2 orders of magnitude)(Supplementary Table S6). The L4 peak von Mises stress and displacement changed by less than 5%, indicating that the global mechanical response was mainly governed by defect geometry and bone cement augmentation, with limited sensitivity to the assumed stiffness of the defect-filling tissue. Bone, defect-filling tissue, and PMMA were modeled as linear elastic and isotropic materials.

**TABLE 1:** Material parameters of different materials in the finite element model.

Structure	Young’s modulus (MPa)	Poisson’s ratio	k (W·m^-1^·K^−1^)	α (K^−1^)	References
Cortical bone	12,000	0.3	0.64	—	Sciortino et al. (2023), Feldmann et al. (2018)
Cancellous bone	100	0.3	0.42	—	Sciortino et al. (2023), Feldmann et al. (2018)
Cartilage	10	0.4	—	—	Han et al. (2021)
Annulus fibrosus	4.2	0.45	—	—	Sciortino et al. (2023)
Nucleus pulposus	1	0.499	—	—	Sciortino et al. (2023)
Endplate	1,000	0.4	—	—	Han et al. (2021)
Defect-filling tissue	1	0.3	—	—	Galbusera et al. (2018)
PMMA	3,000	0.3	0.2	7.0 × 10^−5^	(Zhou et al., 2025; Soleymani Eil Bakhtiari et al., 2021)
^125^I Ti shell	110,000	0.3	—	—	Niinomi and Nakai (2011)

### 2.2 Construction of simulated osteolytic defect and treatment models

**FIGURE 3:**
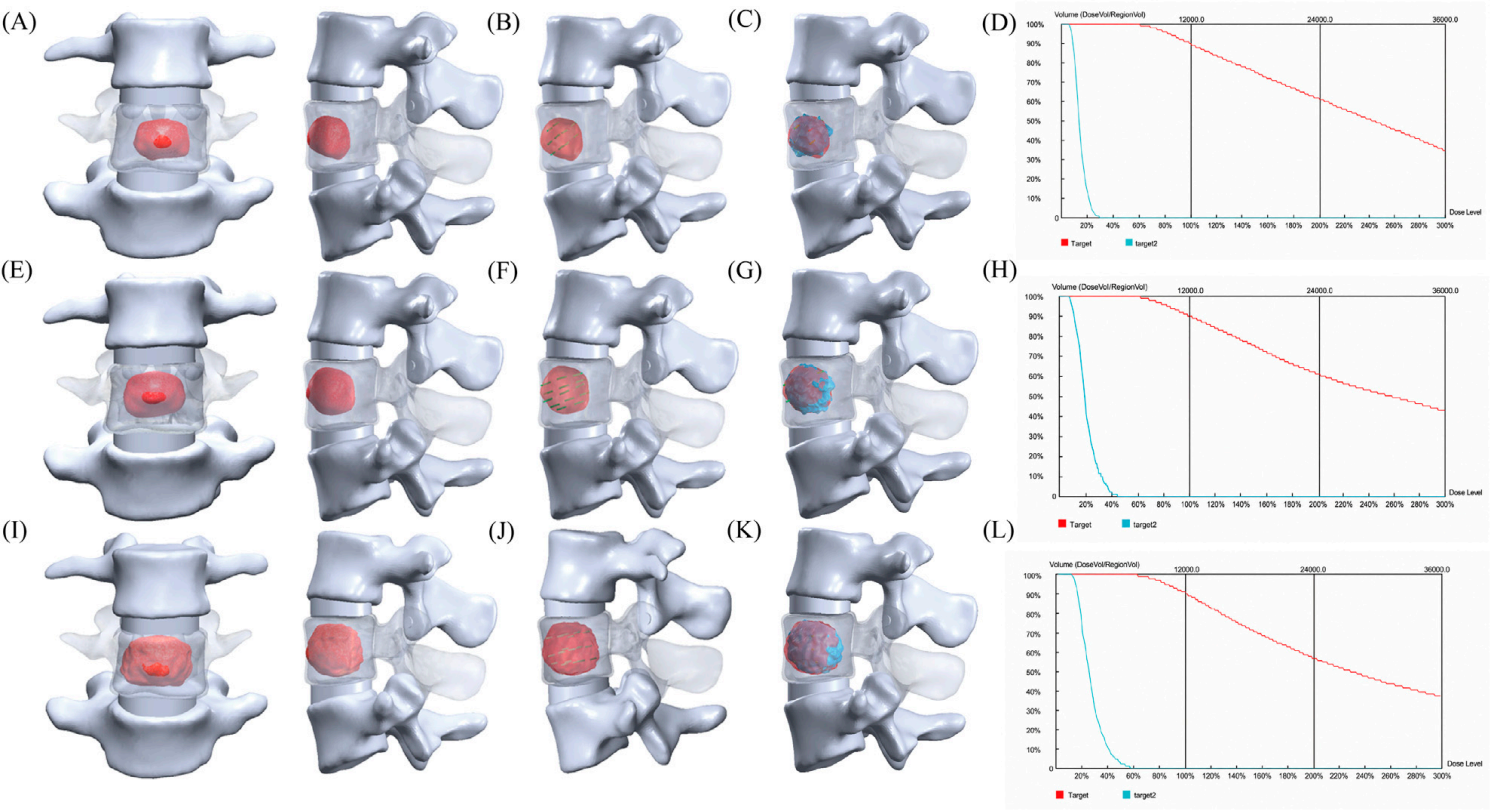
Three-dimensional (3D) models of different simulated osteolytic defect volume groups and corresponding volume-dose histograms (VDHs). **(A–D)** T ≤ 30% group. **(A)** Anterior and lateral views of the osteolytic defect model; **(B)** Lateral view of the ^125^I seed implantation alone model; **(C)** Lateral view of the ^125^I-PVP combination model; **(D)** Corresponding VDH. **(E–H)** 30% < T < 50% group. **(E)** Anterior and lateral views of the osteolytic defect model; **(F)** Lateral view of the ^125^I seed implantation alone model; **(G)** Lateral view of the ^125^I-PVP combination model; **(H)** Corresponding VDH. I–L T ≥ 50% group. **(I)** Anterior and lateral views of the osteolytic defect model; **(J)** Lateral view of the ^125^I seed implantation alone model; **(K)** Lateral view of the ^125^I-PVP combination model; **(L)** Corresponding VDH.

Osteolytic defect model: In SolidWorks, an irregular spherical defect was created in the anterior-middle one-third region of the vertebra to simulate an osteolytic defect, and the defect cavity was generated by Boolean subtraction. The defect-volume fraction (T) was calculated as the ratio of the Boolean-subtracted defect-volume to the original L4 vertebral body volume. The defect geometry was then adjusted iteratively to obtain three predefined defect-volume grades: T ≤ 30%, 30% < T < 50%, and T ≥ 50%. After the defect geometries were finalized, the mesh in the defect region was locally refined to 0.8 mm (Figures 3A,E,I).

^125^I seed implantation alone: OncoSeed 6,711 seeds (0.8 mm × 4.5 mm; activity: 0.8 mCi) were simulated in accordance with the AAPM TG-43 guidelines. Seed positions were generated within the Boolean-subtracted L4 defect cavity according to the modified Paris system, with approximate spacings of 10 mm centrally and 7–8 mm peripherally (Figures 3B,F,J). The seed coordinates were defined in the same global coordinate system as the finite element model and were used for subsequent TG-43 dose calculation. The seed arrangement was checked and adjusted using the volume–dose histogram to ensure 90% prescription isodose coverage of the gross osteolytic defect while respecting spinal cord and nerve-root dose constraints (Zuo et al., 2021) (Figures 3D,H,L).

^125^I seed implantation combined with PVP: Based on the segmented defect cavity, a PMMA fill was modeled to occupy 80%–90% of the cavity volume (Figures 3C,G,K). Under baseline conditions, the bone–cement interface and the interface between necrotic and non-necrotic tissue were prescribed as fully bonded. For structural analysis, the PMMA filler and the surrounding defect region were defined as fully bonded. Frictional contact with relative sliding motion between two contacting surfaces with a friction coefficient of 0.01 was defined between each vertebra/disc surface (Wan et al., 2022).

### 2.3 Sequential coupling of radiation dose, thermal, and mechanical fields

To characterize the multiphysics response to ^125^I seed implantation combined with PVP, we implemented a sequentially coupled finite element workflow in ANSYS (ANSYS Inc., United States) linking the radiation dose, thermal, and mechanical fields. The radiation dose distribution and PMMA polymerization kinetics were computed in Python and mapped onto the finite element mesh via node IDs. Two scenarios were simulated independently: an early post-procedural period (0–30 min; Scenario A), in which only PMMA polymerization heat generation and the resulting thermal stresses were considered, and a late post-procedural period (90 days; Scenario B), in which material properties were updated as a function of the cumulative radiation dose to assess long-term mechanical stability.

For each finite element node, the 90-day cumulative dose D90 was obtained by integrating the TG-43U1 dose-rate over time with ^125^I decay. For material assignment in the structural analysis, nodal D90 values were subsequently averaged at the element level, and elements were classified as necrotic (D90 ≥ 120 Gy), partially damaged (80 ≤ D90 < 120 Gy), or low-dose regions (D90 < 80 Gy) (Wei et al., 2023). The corresponding modulus reductions (Enec = 0.3E, Epart = 0.6E, and a 20% decrease for low-dose bone) were selected as mid-range values within the 20%–40% degradation reported for irradiated bone and further tested in the sensitivity analysis (Mansor et al., 2023). In representative models, TG-43U1 dose maps were also benchmarked against a heterogeneous Monte Carlo calculation including bone, PMMA, and titanium, which confirmed that D90 and necrotic volume fractions agreed within ∼10% and preserved the relative trends between treatment strategies (Carlton et al., 2024).

#### 2.3.1 Dose field calculation and heterogeneity evaluation

In this study, analysis of the major structures at each volume level was based on water-equivalent dose distributions calculated using the TG-43U1 formalism. The ^125^I sources were modeled as line sources, and the dose rate was calculated according to:

$$\dot{D}\left(r,\theta \right)=S_{K}\Lambda \frac{G_{L}\left(r,\theta \right)}{G_{L}\left(r_{0},\theta_{0}\right)}g_{L}\left(r\right)F\left(r,\theta \right)$$

where Ḋ(r,θ) is the dose rate at the calculation point located at polar coordinates (r,θ) relative to the center and longitudinal axis of the ^125^I seed; S_K_ is the air-kerma strength of the source; Λ is the dose-rate constant; G_L_ (r,θ) is the line-source geometry function; G_L_ (r_0_,θ_0_) is the geometry function at the reference point, with r_0_ = 1 cm and θ_0_ = 90°; g_L_(r) is the radial dose function; and F (r,θ) is the two-dimensional anisotropy function. For multiple implanted seeds, the total dose rate at each finite element node was obtained by summing the dose-rate contributions from all seeds. Dosimetric parameters (dose-rate constant, radial dose function, and anisotropy function) were derived from the TG-43U1 consensus data for model 6,711 seeds (Kalinowski and Enger, 2024). Considering the 59.4-day half-life of ^125^I, the 90-day cumulative dose was obtained by integrating the exponentially decaying dose rate over time (Di et al., 2024). The cumulative D90 was first calculated at each finite element node. For material degradation in the structural analysis, nodal D90 values were averaged at the element level and then used to classify elements as necrotic (D90 ≥ 120 Gy), partially damaged (80 ≤ D90 < 120 Gy), or low-dose bone tissue (D90 < 80 Gy). To assess the influence of tissue heterogeneity (bone, PMMA, and titanium shell) on the dose distribution, additional CT-based heterogeneous Monte Carlo dose calculations were performed in representative models (Kim and Enger, 2024), including ^125^I implantation alone for the T ≤ 30% group and ^125^I combined with PVP for the 30% < T < 50% and T ≥ 50% groups. Monte Carlo transport was carried out with an EGSnrc-based code using a voxelized phantom derived from the planning CT, in which cortical/cancellous bone, PMMA, titanium encapsulation, and soft tissue were assigned distinct mass density and elemental compositions (Kalinowski and Enger, 2024).

#### 2.3.2 Thermal field

The exothermic polymerization of PMMA was modeled using an Arrhenius-type kinetic formulation, with a total heat release of approximately 120 kJ/kg, consistent with differential scanning calorimetry (DSC) data reported in the literature (Ramanathan et al., 2024; Chen Y. C. et al., 2024; Tanvir et al., 2025). The kinetic parameters were adopted from previous PMMA studies (pre-exponential factor A≈1.0 × 10^7^ s^-1^, activation energy E_a_ ≈ 80 kJ/mol, reaction order ≈1.5) (Yin et al., 2025). The heat conduction equation included a Pennes-type perfusion term in cancellous bone and surrounding soft tissues, with blood temperature set to 37 °C. Heat loss at the outer vertebral surface was represented by an effective convection boundary condition with a baseline convective heat transfer coefficient h = 10 W m^-2^·K^−1^ (Kniha et al., 2022; Chen Y. C. et al., 2024). Because the vertebra is surrounded by soft tissues rather than exposed to ambient air, this coefficient was used as a lumped representation of net heat exchange with adjacent tissues, including perfusion-related cooling. To address uncertainty, h and the perfusion rate ω were systematically perturbed in the sensitivity analysis.

The thermal conductivity and specific heat of bone tissue and PMMA were defined as temperature-dependent, piecewise functions. Thermal damage was assessed using two complementary criteria: (i) an empirical threshold of temperature >50 °C sustained for ≥1 min, and (ii) the Arrhenius damage integral with Ω ≥ 1 (Chen Y. C. et al., 2024). For each node, time-resolved temperature histories and peak temperatures were recorded and subsequently used to evaluate temperature-induced thermal strains and stresses in the immediate post-procedural condition. The thermal damage volume fraction was defined as the ratio of thermally injured elements to all finite elements within a local region of interest encompassing the PMMA fill, the defect cavity, and the immediately adjacent cancellous bone of L4, rather than the entire vertebral body (Chen Y. C. et al., 2024). Thermally injured elements were used only to calculate the thermal damage volume fraction and were not assigned an additional stiffness-reduction factor in the baseline thermo-mechanical analysis. Elements satisfying either criterion were labeled as thermally injured and used to calculate the thermal damage volume fraction. In the baseline thermo-mechanical analysis, this label was not used to reduce the elastic modulus of the element. Instead, the transient temperature field was applied as a nodal thermal load to calculate thermal strains and stresses. Thus, thermal damage was treated as a thermal exposure metric rather than an on/off material degradation rule.

#### 2.3.3 Field data mapping and material degradation

After meshing in ANSYS, the same structural mesh was retained across all scenarios to enable one-to-one field transfer. The nodal coordinate list of the structural mesh (Node ID, x, y, z; global coordinate system, mm) was exported via an APDL workflow and used as the indexing table. In Python, TG-43 dose-rate contributions from all ^125^I seeds were evaluated at each exported node coordinate and integrated over time with physical decay to obtain the 90-day cumulative dose (Dose 90d, Gy). For cement augmentation cases, PMMA polymerization was simulated to generate a transient nodal temperature history T(t), and the peak temperature field over 0–30 min was extracted (Tmax, °C). Dose and temperature fields were saved as node-wise ASCII tables in a (Node ID, Value) format and re-imported into ANSYS using APDL read commands; the external fields were then mapped one-to-one onto the same finite element mesh by Node ID without geometric interpolation. Temperature was applied as a nodal body load (BF, TEMP) for the immediate post-procedural thermo-mechanical analysis (Scenario A), and thermal strains/stresses were computed using the prescribed thermal expansion coefficients. No elastic-modulus reduction was assigned solely on the basis of the thermal damage label. For the long-term analysis (Scenario B), nodal Dose 90d values were converted to element-wise dose using the element nodal-average; elements were then classified by the predefined dose thresholds, and elastic moduli were updated according to the dose-based modulus-reduction scheme. Mapping fidelity was assessed by (i) verifying node counts after import, (ii) spot-checking randomly selected nodes for numerical consistency, and (iii) confirming agreement of spatial gradients/hotspot locations via dose/temperature contour visualization.

Radiation-induced degradation of bone mechanical properties has been reported in experimental and animal studies, with reductions commonly on the order of tens of percent for high-dose exposures, although the magnitude varies with species, dose/fractionation, and post-irradiation time (Emerzian et al., 2023; Carney et al., 2024). Given the limited human vertebra-specific evidence under continuous low-dose-rate ^125^I brachytherapy, we used a pragmatic, dose-stratified modulus reduction scheme to represent potential local softening rather than to define human-specific constants. Because most available quantitative data are derived from external beam radiotherapy or animal models with different dose-rate and remodeling kinetics, these reduction factors were used as a trend-capturing surrogate rather than a validated constitutive law for human vertebrae under low-dose-rate (LDR) exposure. Based on the 90-day cumulative dose, tissues were categorized as necrotic (D 90d ≥ 120 Gy), partially damaged (80 ≤ D 90d < 120 Gy), or lower-dose bone (D 90d < 80 Gy), with elastic moduli set to E nec = 0.3 E, E part = 0.6 E, and a uniform 20% reduction for lower-dose bone as a conservative approximation.

Geometrically nonlinear structural analyses were conducted, and moderate numerical damping was introduced under conditions of pronounced material softening to improve convergence. The temperature field was considered only in the immediate post-procedural setting (Scenario A) to evaluate thermal stresses and thermal damage (Yin et al., 2025; Chen Y. C. et al., 2024). Scenario B was interpreted as primarily governed by dose-driven modulus degradation, with thermally induced effects playing a secondary role at the 90-day timescale. To assess the impact of the degradation assumption on mechanical response, parameter-perturbation scenarios were defined around the baseline model (E nec = 0.3 E, E part = 0.6 E, and 20% softening for lower-dose bone). The ratios E nec/E part were varied to 0.4E/0.7E and 0.2E/0.5E, and the softening of lower-dose bone was adjusted to 10% and 30% (Doulgeris et al., 2023). Structural analyses were repeated for the three volumetric grading levels, and maximum L4 displacement, maximum von Mises stress, and their relative trends across treatment strategies were compared.

### 2.4 Evaluation metrics and sensitivity analysis

To evaluate model robustness and parametric uncertainty, a sensitivity analysis was performed on the worst-case defect category (T ≥ 50%) under flexion loading, using the baseline ^125^I seed implantation combined with PVP model, which incorporates key uncertainties in cement mechanics and PMMA polymerization heat transfer. Evaluation metrics included: Maximum von Mises stress, maximum displacement of the L4 vertebral body, peak temperature and temperatures at key intraspinal canal locations, volume fraction of thermally damaged tissue, and proportion of thermally induced stress relative to total stress. The present study did not calculate vertebral failure load or apply an explicit failure criterion. Mechanical stability was evaluated by comparing maximum L4 displacement and maximum von Mises stress under physiological loading. The 1.9% apparent strain method was not used because this L3–L5 segmental model was not designed for isolated-vertebra axial compression-to-failure analysis (Gibson et al., 2025). Investigated parameters included: PMMA elastic modulus (±20%), bone density (corresponding to a ±30% variation in elastic modulus E), necrosis and partial-damage dose thresholds (±10%), total polymerization heat release (±15%), convective heat transfer coefficient (h, ±50%), and perfusion rate (ω, ±30%). Sensitivity was quantified by comparing the relative deviations of each output metric from the baseline across the parameter-perturbation scenarios, with particular attention to peak temperature and the thermally damaged volume fraction in the T ≥ 50% models.

## 3 Results

This study reports results for two scenarios: the long-term mechanical scenario at 90 days post-procedure (Scenario B) and the immediate post-procedure thermal scenario (Scenario A). Results for Scenario B (long-term biomechanical response) are presented first, followed by the immediate thermal effects (Scenario A). Thermal results were used only to aid interpretation of long-term mechanical trends.

### 3.1 Model validation and comparison with published ROM data

**FIGURE 4:**
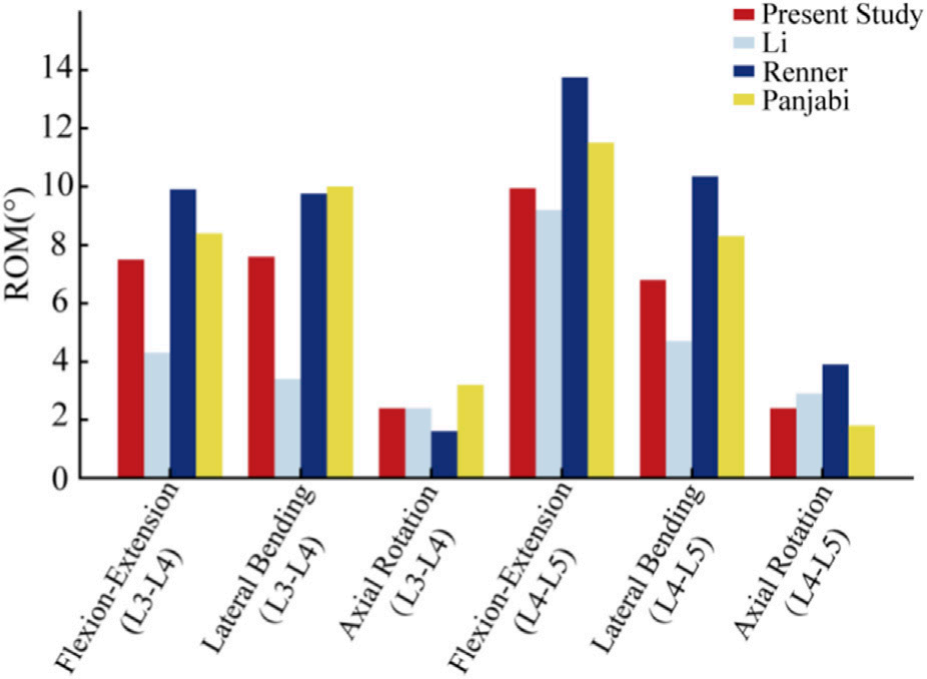
ROM of the L3–L5 segment in the intact finite element model was compared with previously reported data under a pure 7.5 N m moment (with a 500 N compressive preload when applicable).

Mesh convergence was evaluated for the coupled mechanical, thermal, and dosimetric outputs using the representative T ≥ 50% model treated with ^125^I seed brachytherapy combined with PVP under flexion loading. The baseline 1.0 mm mesh was compared with coarse 1.5 mm and refined 0.8 mm meshes. Compared with the refined mesh, all evaluated mechanical, thermal, and dosimetric outputs showed relative differences below 5% (Supplementary Table S7), indicating that the baseline mesh was sufficient for the subsequent coupled analyses. Because no physical experimental setup was used in this study, model validation was performed by comparing the ROM of the intact L3–L5 model with previously reported experimental and finite element data under comparable loading conditions. The ROM of the intact model under a pure moment of 7.5 N m in flexion, extension, lateral bending, and axial rotation fell within the previously reported ranges (Li et al., 2009; Renner et al., 2007; Panjabi et al., 1994)(Figure 4).

### 3.2 Scenario B: long-term biomechanical response at 90 days

#### 3.2.1 T ≤ 30% (small defect volume)

**FIGURE 5:**
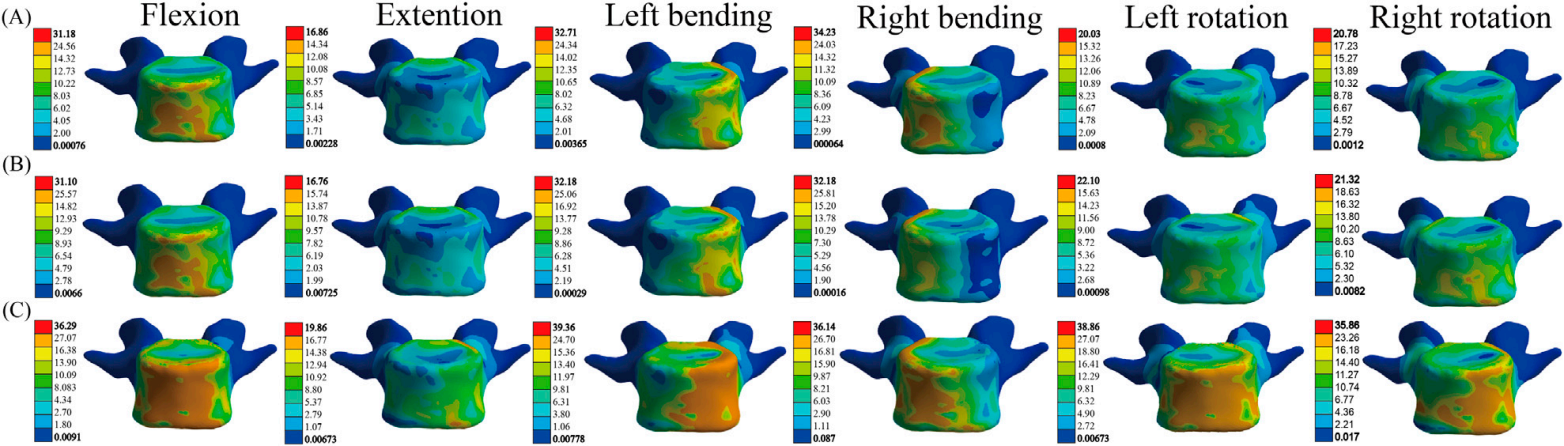
Scenario B (90-day) biomechanical responses of the L4 vertebra in the T ≤ 30% group. The maximum von Mises stress distributions under flexion, extension, left/right lateral bending, and left/right axial rotation are shown for **(A)** osteolytic defect model **(B)** ^125^I seed implantation alone, and **(C)** ^125^I-PVP combination group. In this low-volume group, ^125^I seed implantation alone produced negligible changes compared with the osteolytic defect model, whereas cement augmentation increased maximum von Mises stress across all motion directions, indicating stress concentration without a clear biomechanical benefit.

In the T ≤ 30% group, the maximum von Mises stresses of the osteolytic defect model under flexion, extension, left lateral bending, right lateral bending, left rotation, and right rotation were 31.18, 16.86, 32.71, 34.23, 20.03, and 20.78 MPa, respectively. After ^125^I seed implantation, these values changed minimally to 31.10, 16.76, 32.18, 32.18, 22.10, and 21.32 MPa, respectively. Following PMMA augmentation, the corresponding maximum von Mises stresses rose to 36.29, 19.86, 39.36, 36.14, 38.86, and 35.86 MPa, showing a clear upward trend (Figure 5). Notably, in small-volume simulated defects (T ≤ 30%), PVP did not provide a measurable mechanical benefit but increased local stress, indicating potential stress concentration associated with stiffness mismatch. Overall, ^125^I implantation alone preserved vertebral stability, whereas routine PMMA augmentation increased the maximum von Mises stress without improving long-term stability metrics.

#### 3.2.2 30% < T < 50% (moderate defect volume)

**FIGURE 6:**
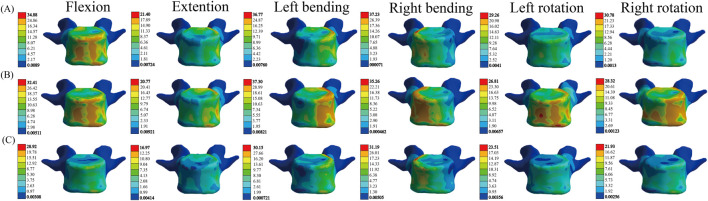
Scenario B (90-day) biomechanical responses of the L4 vertebra in the moderate defect-volume group (30% < T < 50%). The maximum von Mises stress in L4 under flexion, extension, left/right lateral bending, and left/right axial rotation is compared among **(A)** osteolytic defect model **(B)** ^125^I seed implantation alone, and **(C)** ^125^I-PVP combination group. Cement augmentation reduced peak stress and displacement compared with the other two models.

In the 30% < T < 50% group, the maximum von Mises stresses of the osteolytic defect model were 34.88, 21.40, 36.77, 37.23, 29.26, and 30.78 MPa under flexion, extension, left lateral bending, right lateral bending, left axial rotation, and right axial rotation, respectively. Following ^125^I seed implantation, these values changed to 32.41, 20.77, 37.30, 35.26, 26.81, and 28.32 MPa, showing minimal changes. After bone cement augmentation, flexion displacement was reduced from 8.20 mm to 4.36 mm, and extension displacement from 2.40 mm to 1.70 mm. The corresponding maximum stresses under flexion, extension, left lateral bending, right lateral bending, left axial rotation, and right axial rotation were reduced to 28.92, 16.97, 30.15, 31.19, 23.51, and 21.93 MPa, respectively (Figure 6). Thus, in the moderate simulated osteolytic defect group, the combined strategy of ^125^I seed implantation and PVP substantially improved displacement control and reduced stress compared with ^125^I implantation alone.

#### 3.2.3 T ≥ 50% (large defect volume)

**FIGURE 7:**
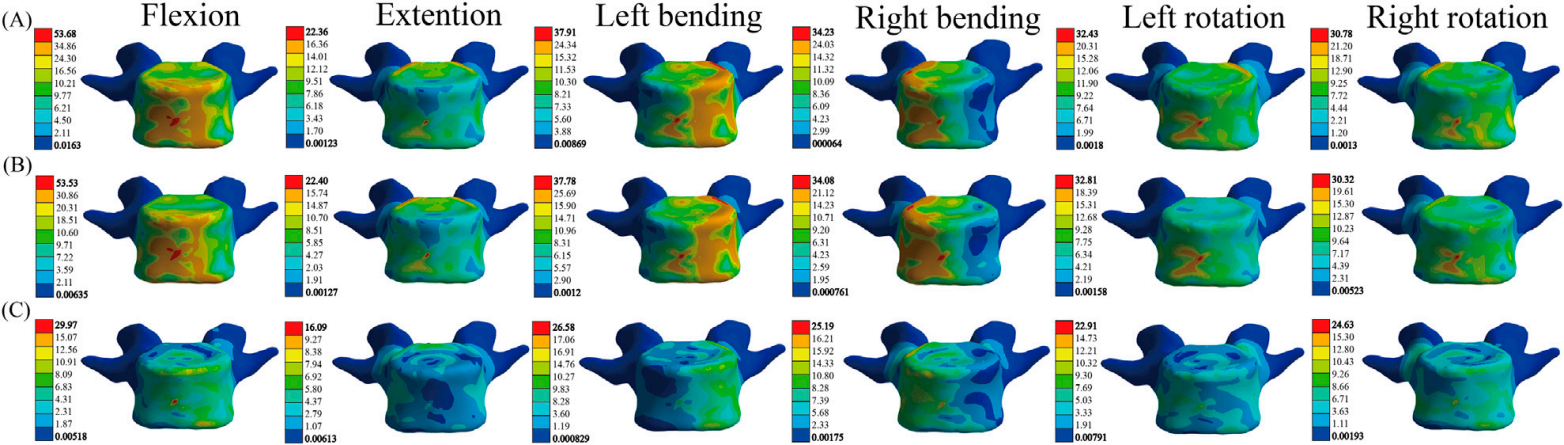
Scenario B (90-day) biomechanical responses of the L4 vertebra in the large defect-volume group (T ≥ 50%). The maximum von Mises stress in L4 under flexion, extension, left/right lateral bending, and left/right axial rotation is compared among **(A)** osteolytic defect model **(B)** ^125^I seed implantation alone, and **(C)** ^125^I-PVP combination group. Cement augmentation markedly reduced peak stress and displacement relative to the other two models.

In the T ≥ 50% group, the maximum von Mises stresses of the osteolytic defect model were 53.68, 22.36, 37.91, 34.23, 32.43, and 30.78 MPa under flexion, extension, left lateral bending, right lateral bending, left rotation, and right rotation, respectively. After standalone ^125^I seed implantation, these values were recorded as 53.53, 22.40, 37.78, 34.08, 32.81, and 30.32 MPa. After PMMA augmentation, flexion displacement decreased from 8.81 mm to 4.76 mm, and rotational displacement from 5.96 mm to 2.30 mm. The maximum von Mises stress under flexion decreased from 53.68 MPa to 29.97 MPa, and the peak stresses under extension, left lateral bending, right lateral bending, left rotation, and right rotation were reduced from 22.36, 37.91, 34.23, 32.43, and 30.78 MPa to 16.09, 26.58, 25.19, 22.91, and 24.63 MPa, respectively (Figure 7). Therefore, in large-volume simulated defects, the combination of ^125^I seed implantation and PVP substantially restored biomechanical performance, with cement-mediated reinforcement dominating long-term stability improvements.

### 3.3 Scenario A: immediate thermal effects and postoperative safety (0–30 min)

**FIGURE 8:**
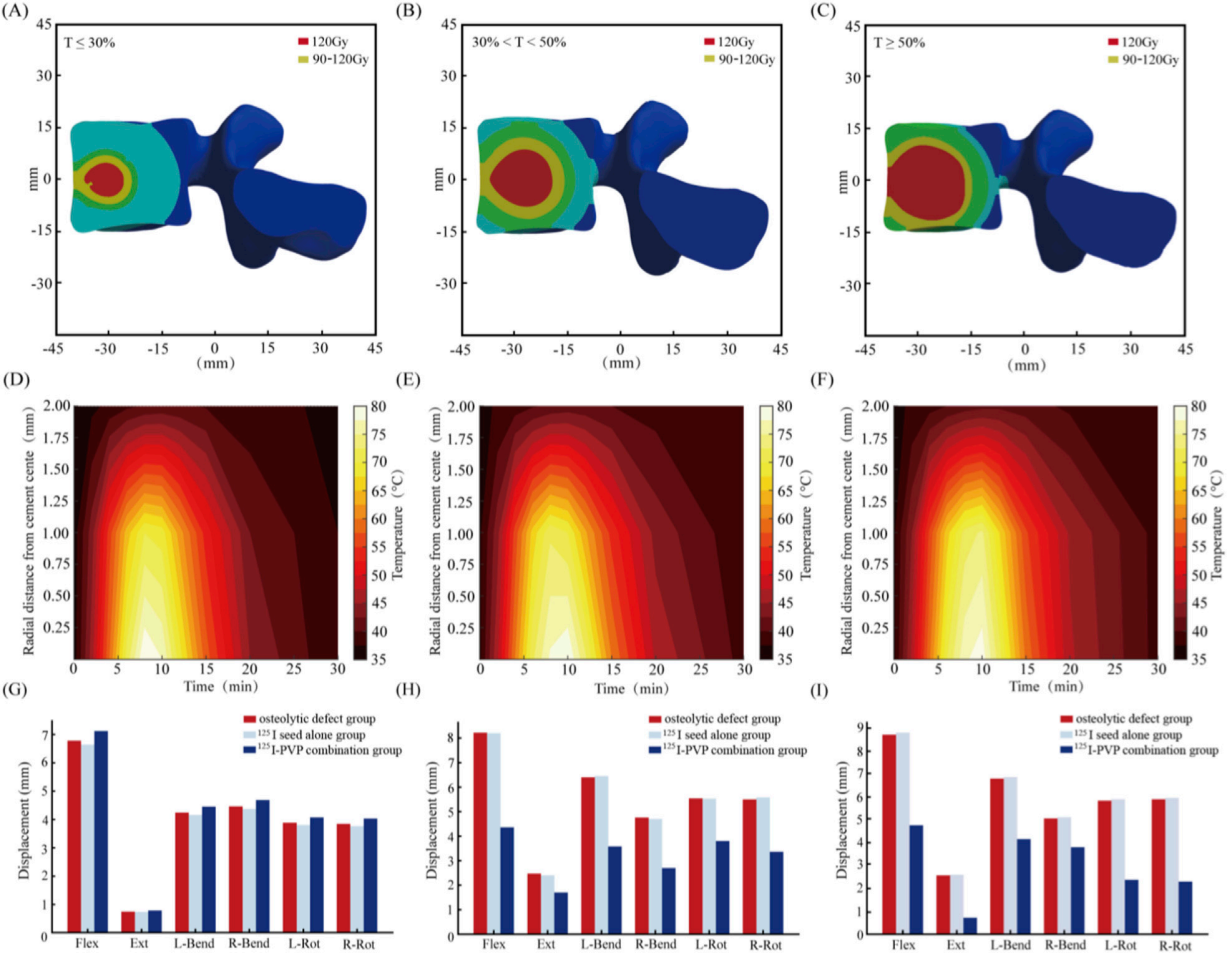
Distributions of prescription-dose coverage, bone cement polymerization temperature, and biomechanical properties. **(A–C)** Prescription-dose coverage volume fraction distribution: A T ≤ 30%; B 30% < T < 50%; C T ≥ 50%. **(D–F)** Bone cement polymerization temperature distribution: D T ≤ 30%; E 30% < T < 50%; F T ≥ 50%. **(G–I)** Maximum L4 displacement distribution: G T ≤ 30%; H 30% < T < 50%; I T ≥ 50%.

Thermal damage and peak temperatures are model-dependent, with a focus on relative differences across volumetric grades (Figure 8). Thermal effects of PMMA polymerization were quantified for cement-augmented models during the immediate post-procedural period (0–30 min) and are summarized by defect-volume grade. The T ≤ 30% group exhibited a peak temperature of approximately 74 °C with a thermal damage volume fraction of ∼9%. In the 30% < T < 50% group, the peak temperature was approximately 80 °C, with a thermal damage volume fraction of ∼15% and a thermal stress contribution of ∼15%. In the T ≥ 50% group, the larger cement fill volume increased the peak PMMA temperature to approximately 84 °C, yielding a thermally damaged volume fraction of approximately 25% and a thermal stress contribution of approximately 16%. Overall, thermal stress accounted for approximately 10%–20% of the total von Mises stress. Importantly, spinal canal temperatures remained below the commonly cited thermal injury threshold of 43 °C–45 °C in all groups, suggesting a low thermal risk to neural structures under the modeled conditions.

### 3.4 Sensitivity analysis

Sensitivity analyses (T ≥ 50% group) showed that uncertainties in mechanical parameters mainly affected stress with limited influence on displacement. A ±20% change in bone cement elastic modulus led to an approximately −30%/+23% change in maximum stress, whereas displacement remained within ∼±10%. A ±30% variation in BMD altered the Maximum von Mises stress by approximately by ∼+20.3%/−16% and displacement by ∼+15%/−10%, with negligible effects on temperature and thermal injury. In contrast, thermal parameters predominantly governed temperature-related outputs while leaving mechanical responses essentially unchanged: a ±15% change in polymerization heat release induced a ∼−8/+10 °C shift in peak temperature and a ∼-24.8%/+30% change in thermal damage volume fraction. A ±50% variation in the convective heat transfer coefficient (h) changed the peak temperature by −6.0% to +8.3% and the thermal damage volume fraction by −24.8% to +24.8%. For a ±30% variation in perfusion rate (ω), a 30% decrease in ω increased peak temperature and thermal injury by ∼7.1% and 20%, while a 30% increase decreased them by ∼4.8% and 15.2%; across these perturbations, the maximum stress and displacement of the L4 segment remained largely unchanged.

For dose-related uncertainty, varying the dose threshold by ±10% changed the maximum stress by approximately −5.8% to +7.9%, but shifted the necrotic-volume fraction from 77% to 93% (Table 2). The thermal damage fractions estimated using the empirical threshold method and the Arrhenius damage integral were also compared across the three defect-volume grades (Table 3). Results for the other two defect-volume grades are reported in Supplementary Tables S1, S2. Under perturbations of dose–material degradation parameters (Enec: 0.2E–0.4E; Epart: 0.5E–0.7E; and 10%–30% softening of low-dose bone tissue), variations in the maximum displacement and stress of the L4 vertebra across all three volumetric grades were confined within ∼10% (Supplementary Tables S31–S5), and the relative ranking between ^125^I seed implantation alone and ^125^I seed brachytherapy combined with PVP remained unchanged. TG-186–based corrections further indicated that, for simulated defects with T ≤ 30% without PMMA, TG-43U1 overestimated D90 and the necrotic volume fraction by ∼5–7% relative to Monte Carlo calculations (Table 4), whereas for simulated defects with 30% < T < 50% and T ≥ 50% treated with PMMA, Monte Carlo predicted D90 and necrotic volume fractions ∼4–8% higher than TG-43U1 (Table 5). When these dose-driven material degradation fields were incorporated into the structural analysis, changes in the maximum von Mises stress and displacement remained within ∼10%, and the mechanical advantage of ^125^I seed brachytherapy combined with PVP over ^125^I seed implantation alone was preserved across all defect-volume categories. The relative trends between treatment strategies remained consistent across parameter variations, supporting the robustness of the findings.

**TABLE 2:** Sensitivity analysis of the T ≥ 50% group.

Parameter perturbation	Max VMS (MPa)	Max L4 displacement (mm)	Peak temp (°C)	Spinal canal key point temp (°C)	Thermal damage vol fraction (%)	Thermal stress ratio (%)	Thermal stress (MPa)	Mechanical stress (MPa)	Element-wise necrotic-volume fraction after dose averaging (%)
Baseline	29.97	4.76	84	42.0	25.0	16	4.92	24.98	85
Bone cement modulus: −20%	20.98	5.23	84	42.0	25.0	18	3.76	17.18	85
Bone cement modulus: +20%	36.85	4.29	84	42.0	25.0	21	7.64	28.89	85
Bone mineral density: −30%	36.06	5.47	84	42.0	25.0	20	7.31	28.74	85
Bone mineral density: +30%	25.18	4.30	84	42.0	25.0	18	4.52	20.34	85
Polymerization exotherm: −15%	29.97	4.76	76	41.2	18.8	17	5.08	23.59	85
Polymerization exotherm: +15%	29.97	4.76	94	43.0	32.5	26	7.81	22.56	85
Convective heat transfer coefficient (h): −50%	29.97	4.76	91	42.7	31.2	24	7.21	22.92	85
Convective heat transfer coefficient (h): +50%	29.97	4.76	79	41.5	18.8	17	5.08	23.59	85
Perfusion rate (ω): −30%	29.97	4.76	90	42.6	30.0	24	7.21	22.92	85
Perfusion rate (ω): +30%	29.97	4.76	80	41.6	21.2	17	5.08	23.59	85
Dose threshold: −10%	32.34	4.76	84	42.0	25.0	18	5.90	25.18	93
Dose threshold: +10%	28.24	4.76	84	42.0	25.0	18	5.22	21.52	77

Temp = temperature; Vol = volume. Thermal stress ratio (%): Rth = σeq, th/σeq, tot × 100, where σeq, th and σeq,tot denote the von Mises stresses under thermal-only and combined thermo–mechanical loading, respectively. The element-wise necrotic-volume fraction represents the finite element volume fraction classified as necrotic after nodal D90 values were averaged at the element level for dose-driven material degradation.

**TABLE 3:** Comparison of the empirical threshold method and arrhenius thermal damage fraction.

Defect-volume grade	Peak temperature (°C)	Empirical threshold method (T > 50 °C and duration ≥1 min)	Arrhenius damage integral (Ω ≥ 1)	Relative difference
T ≤ 30%	74.2 ± 3.1	0.09	0.10	+11%
30% < T<50%	80.1 ± 3.4	0.15	0.17	+13%
T ≥ 50%	84.0 ± 3.8	0.25	0.22	−12%

**TABLE 4:** Dosimetric comparison for ^125^I seed implantation alone by volume category: TG-43U1 versus heterogeneity-corrected Monte Carlo calculations.

Defect-volume grade	Dose model	D90 (Gy)	ΔD90 (%)	Dmean (Gy)	ΔDmean (%)	V ≥ 120Gy (%)	ΔV≥120Gy (%)
T ≤ 30%	TG-43	120.0	–	303.6	–	89	–
​	Monte Carlo	113.1	−5.80	292.6	−3.60	84	−5.6
30% < T < 50%	TG-43	120.0	–	300.7	–	91	–
​	Monte Carlo	113.1	−5.80	291.2	−3.20	83	−8.8
T ≥ 50%	TG-43	120.0	–	298.5	–	90	–
​	Monte Carlo	113.1	−5.80	287.4	−3.70	84	−6.7

**TABLE 5:** Dosimetric comparison of ^125^I seed brachytherapy combined with PVP across defect-volume categories: TG-43U1 versus heterogeneity-corrected Monte Carlo calculations.

Defect-volume grade	Dose model	D90 (Gy)	ΔD90 (%)	Dmean (Gy)	ΔDmean (%)	V ≥ 120Gy (%)	ΔV≥120Gy (%)
T ≤ 30%	TG-43	120.0	–	298.1	–	92	–
​	Monte Carlo	125.3	+4.40	309.9	+4.00	94	+2.17
30% < T < 50%	TG-43	120.0	–	306.0	–	90	–
​	Monte Carlo	125.1	+4.30	316.4	+3.40	92	+2.22
T ≥ 50%	TG-43	120.0	–	300.2	–	89	–
​	Monte Carlo	125.1	+4.30	311.6	+3.80	92	+3.37

## 4 Discussion

The management of osteolytic spinal metastases primarily aims to achieve pain relief, local tumor control, and preservation or restoration of mechanical stability. In current practice, decisions on whether to perform ^125^I seed implantation alone or to combine with PVP remain largely experience-based and lack quantitative support. Existing spinal instability scoring systems, such as the Spinal Instability Neoplastic Score (SINS), provide useful guidance but incorporate limited quantitative information on lesion volume and its mechanical consequences. In this study, we combined a defect-volume stratification scheme with a sequentially coupled dose–thermal–mechanical finite element framework to elucidate the underlying mechanisms and to compare, across different degrees of vertebral compromise, ^125^I seed implantation alone with ^125^I seed brachytherapy combined with PVP. These defect-volume thresholds are intended to describe model-observed transitions and should not be interpreted as prescriptive clinical cut-offs.

### 4.1 Role and controversy of ^125^I seed implantation and PVP in the treatment of spinal metastases

In the context of spinal metastases, the literature is largely consistent in supporting the use of PVP (Tomasian et al., 2023). In both benign and malignant vertebral lesions, PVP provides rapid and sustained relief of mechanical pain, improves quality of life, and is less invasive than conventional open decompression and instrumentation (Korovessis et al., 2025). However, increasing evidence from imaging follow-up studies and biomechanical analyses indicates that high-modulus PMMA cement substantially increases segmental stiffness and may redistribute loads to adjacent vertebrae, which is a plausible contributor to adjacent-level fracture risk in selected patients (Kim et al., 2025b). Concerns are particularly relevant when lesion burden is small and there is no overt instability: in such cases, prophylactic cement augmentation performed primarily for analgesia may represent overtreatment if mechanical benefit is limited and additional risks (cement leakage, stress concentration, thermal injury, and adjacent fracture) are introduced.

Clinical series evaluating combined ^125^I seed implantation and PVP often report improved pain relief and local control compared with PVP alone (Xu et al., 2023), but many studies do not stratify patients by lesion volume, defect topology, or structural integrity (e.g., cortical/endplate continuity, pedicle/posterior element involvement). Consequently, the clinically actionable question—under which mechanical conditions ^125^I implantation alone is adequate and under which conditions adjunctive PVP is justified—remains insufficiently quantified. This gap motivates a mechanistic, volume-informed framework rather than reliance on collapse-only or fracture-only stratification.

Defect Volume–Based Stratification and Vertebral Instability Mechanisms: Complementing Existing Scoring Systems.

Previous experimental and finite element studies consistently indicate that osteolytic defect volume is a key structural determinant of vertebral instability risk (Soltani et al., 2024; Palanca et al., 2023). When the defect volume approaches ∼30% of the total vertebral body volume, the VB/VH ratio and surface strain shift from a slowly increasing, quasi-linear pattern to a distinctly nonlinear response, and the failure initiation zone relocates to the defect margins. When defect volume exceeds ∼50%, geometric continuity is markedly compromised and physiologic loading can precipitate collapse (Kim et al., 2025a). Accordingly, the volumetric stratification adopted in this study (T ≤ 30%, 30% < T < 50%, T ≥ 50%) has a clear biomechanical rationale.

The present findings are also consistent with the broader CT/DVC literature showing that vertebral mechanical response is governed not only by defect volume but also by lesion morphology, location, and local microstructural changes. Palanca, Dall’Ara, and colleagues demonstrated that DVC can reveal internal strain concentrations and failure localization in metastatic vertebrae (Palanca et al., 2016; Cavazzoni et al., 2023), while recent CT-based subject-specific FE studies have used DVC measurements as experimental benchmarks for model validation (Garavelli et al., 2025). Compared with these studies, the present work does not aim to reproduce patient-specific metastatic vertebrae or DVC-measured strain fields. Instead, it uses idealized simulated osteolytic defects to isolate the relative effects of ^125^I implantation, PMMA augmentation, radiation-induced material degradation, and polymerization heat on vertebral stability. Therefore, our results should be interpreted as treatment-related biomechanical trends that complement, rather than replace, patient-specific CT/DVC-based validation studies (Garavelli et al., 2025; Gibson et al., 2025).

Importantly, however, the 30% and 50% thresholds in this study should be interpreted as model-derived transition trends rather than absolute clinical cut-off values. In practice, the effective instability transition is expected to vary with lesion topology and location (e.g., anterior column versus posterior element involvement, pedicle compromise, endplate/cortical shell disruption, eccentric defects), baseline bone quality (BMD/osteoporosis), and neurological status/epidural disease, which may necessitate decompression or stabilization independent of volumetric grade (Alkalay and Harrigan, 2016; Palacio Giraldo et al., 2025). Therefore, volume fraction should be viewed as a quantitative risk descriptor that can shift depending on patient-specific anatomy and disease pattern, rather than a universal boundary. Because this study is based on a single healthy male L3–L5 geometry with simulated osteolytic defects, patient-level heterogeneity is not represented and the exact transition points (≈30% and ≈50%) should be interpreted as trend-based rather than universally applicable.

Within this context, quantitative osteolysis volume could complement SINS by adding a mechanically grounded continuous variable to improve discrimination among patients with similar radiographic collapse but substantially different lesion burdens (Palacio Giraldo et al., 2025). Such volume metrics can be extracted from routine CT using semi-automated segmentation, which may facilitate integration into clinical workflow and SINS-like decision frameworks. A practical next step is to evaluate whether CT-derived volumetric measures, incorporated as a continuous or discretized modifier alongside existing SINS components, improve prediction of progressive collapse, guide augmentation selection, and stratify post-procedural failure risk in multicenter cohorts.

Within this single-subject FE framework, the adopted dose–modulus mapping should be interpreted as a trend-capturing surrogate rather than a validated constitutive law for human vertebrae under low-dose-rate ^125^I brachytherapy. Human-specific time-dependent remodeling and microdamage accumulation under continuous LDR exposure remain insufficiently quantified (Emerzian et al., 2023; Carney et al., 2024). Nevertheless, the sensitivity analyses demonstrated that the principal conclusion—mechanical benefit of adding PVP for T > 30% and limited benefit for T ≤ 30%—was robust to plausible variations in the degradation parameters, supporting the use of this model as a comparative decision-support framework rather than an absolute predictor of failure (Soltani et al., 2024).

### 4.2 Multiphysics perspective: interactions between radiation, polymerization heat, and mechanical reinforcement

Animal studies and *in vitro* experiments on irradiated bone have demonstrated that radiation can reduce bone material stiffness by approximately 20%–40% (Emerzian et al., 2023; Carney et al., 2024), affecting not only the target region but also surrounding bone. This implies that ^125^I implantation—while delivering a therapeutic radiation dose—may concurrently weaken local structural competence via dose-driven degradation, particularly when larger irradiated volumes are present (Cao et al., 2025). The present results provide a direct mechanistic link to this “double-edged sword” concept: ^125^I implantation alone did not improve stability once defect volume exceeded ∼30%, because brachytherapy does not restore load-bearing continuity and, in Scenario B, the dose-updated material fields introduce stiffness reductions within necrotic/partially damaged regions and adjacent irradiated bone. For small simulated defects (T ≤ 30%), geometric integrity is largely preserved, so the net mechanical effect remains modest, consistent with negligible changes in stress and displacement after ^125^I alone. In contrast, for T > 30% the vertebra enters a nonlinear risk regime governed by architectural compromise; under these conditions, dose-driven softening can offset any indirect mechanical benefit from radiation-mediated local control, yielding no measurable improvement in displacement or stress. By comparison, PMMA augmentation provides an immediate stiffness restoration and load redistribution that dominates the mechanical response, explaining the marked stabilization observed for ^125^I + PVP in the 30%–50% and ≥50% groups.

Regarding thermal effects, prior measurements and simulations indicate that PMMA polymerization can generate 70 °C–90 °C at the bone–cement interface, sufficient for focal osteonecrosis, whereas spinal canal temperatures generally remain below commonly used neural injury thresholds. In our models, peak cement-adjacent temperatures and thermal injury fractions were consistent with ranges reported in previous studies, while key spinal canal locations remained below neural injury thresholds; thermal stress contributed a limited proportion of total von Mises stress and did not overturn the dominant stiffness-based reinforcement effect of cement. These findings support a clinically relevant separation of timescales: polymerization heat is an acute, minute-scale event relevant to immediate safety, whereas dose-driven degradation unfolds over weeks to months and governs longer-term mechanical competence at 90 days or beyond (Chen Y. C. et al., 2024).

To facilitate translational interpretation, the predicted thermal injury fractions should be contextualized as follows. The reported thermally damaged volume fractions (≈8–25%) represent localized injury within a region of interest surrounding the PMMA fill and the immediately adjacent trabecular bone, rather than global necrosis of the vertebral body. Clinically, such interface-confined thermal injury is most plausibly associated with focal trabecular osteonecrosis and microvascular disruption near the cement margin. Whether this focal injury progresses to clinically detectable osteonecrosis, influences pain trajectory, or contributes to delayed mechanical degradation depends on patient-specific healing/remodeling capacity and interface stability, which are not explicitly modeled here. This phenomenon may have bidirectional implications: on the one hand, localized thermal cytotoxicity may contribute to short-term analgesia and local cytotoxic effects; on the other hand, excessive interface necrosis may promote bone resorption, delayed microdamage repair, and potential weakening of the bone–cement interlock over time, which could increase the risk of progressive deformation or adjacent-level stress transfer in susceptible patients. Importantly, our simulations indicate that spinal canal temperatures remain below commonly cited neural injury thresholds, supporting short-term thermal safety for neural structures under the modeled conditions. However, the present model does not explicitly simulate post-thermal biological processes (e.g., inflammation, remodeling, osteolysis/osteogenesis balance, or interface micromotion) that determine whether focal necrosis resolves or progresses. Therefore, the thermal injury fractions should be interpreted as a relative, model-derived indicator of interface thermal exposure rather than a direct predictor of clinically observable osteonecrosis progression or long-term mechanical degradation (Anselmetti et al., 2009; Stańczyk and van Rietbergen, 2004). Future work integrating interface remodeling laws, fatigue loading, and longitudinal imaging/clinical outcomes would be needed to link predicted thermal exposure to clinically meaningful endpoints such as pain trajectory, cement loosening, or delayed collapse.

Notably, the clinical principle that local disease control or ablation may leave residual instability risk if structural competence is not addressed is supported by evidence from other minimally invasive local therapies. A recent systematic review focusing on vertebral stability after radiofrequency ablation (RFA) for vertebral metastatic lesions emphasized post-procedural stability as a key consideration and discussed the role of percutaneous vertebroplasty in mitigating fracture and instability risk (Colonna et al., 2023). Together with our findings, this supports a unified perspective: as architectural compromise increases, stabilization strategies (cement augmentation and/or instrumentation, guided by neurological status and overall instability assessment) become increasingly central to achieving durable palliation and function.

### 4.3 Limitations and future directions

This study has several limitations. First, patient-specific metastatic spines were not analyzed; instead, idealized osteolytic defects were artificially introduced into a healthy lumbar spine model and are therefore referred to as simulated osteolytic defect. *In vivo*, metastatic lesions may contain heterogeneous components (solid tumor, fibrosis, residual trabeculae, and reactive sclerosis) that can provide partial structural support; therefore, representing the defect region as a uniform low-stiffness region may overestimate instability in some cases, although it may be a conservative approximation for predominantly lytic lesions. In addition, the model predictions were not validated against experimental full-field strain measurements such as DVC, which has increasingly been used to characterize and validate the mechanics of metastatic vertebrae. Future work should incorporate patient-specific CT/micro-CT-based models and DVC-based experimental validation. Moreover, because neither phantom-based nor phantomless CT calibration was performed, CT-density-based element-wise material mapping was not implemented; homogeneous literature-based properties were assigned to cortical and cancellous bone. Future studies should incorporate validated CT calibration methods to generate spatially heterogeneous, patient-specific material properties. Second, the mechanical evaluation relied on quasi-static physiological loading and did not implement an explicit vertebral failure criterion or failure-load calculation. Thus, the present results should be interpreted as relative stress/displacement responses rather than absolute predictions of vertebral failure load. The 1.9% apparent strain method was not adopted because this study focused on segmental loading responses rather than isolated-vertebra compression-to-failure analysis. Future work should incorporate cyclic loading, nonlinear constitutive laws, progressive axial compression, element-level damage or yielding, cement–bone interface micromotion, and experimental validation to better assess long-term failure risk after augmentation. Third, the dose-driven modulus degradation model (necrotic/partial/low-dose softening) is a simplified phenomenological representation of complex radiobiological processes involving remodeling dynamics, vascular changes, inflammation, and time-dependent healing/degeneration. Although sensitivity analyses suggested that the main stress/displacement trends were robust to plausible parameter ranges, patient-specific nonlinear dose–response relationships and remodeling trajectories should be investigated. In addition, the present model did not explicitly simulate post-thermal biological remodeling, cement–bone interface degradation, or time-dependent stiffness loss caused by thermal osteonecrosis. Therefore, the predicted thermal damage fraction was used as a relative indicator of local thermal exposure rather than as a direct constitutive damage variable. Future studies should incorporate experimentally calibrated thermal damage–dependent material laws and interface remodeling models to evaluate whether localized thermal injury leads to delayed mechanical weakening or cement–bone interlock degradation.

Finally, the present framework was developed from a single-subject geometry and a limited set of lesion morphologies and locations. Extending the model set to osteoporotic bone, alternative lesion topologies (e.g., posterior element or pedicle involvement, endplate disruption, and eccentric defects), and broader patient cohorts will be essential for external validation and for translating the observed volume-based trends into clinically reliable decision support. Future work should validate these trends in multiple patient-specific models spanning vertebral size, BMD, disc degeneration, and diverse lesion morphologies and locations to quantify how much the thresholds may shift across individuals. This could be complemented by cadaveric models with thermocouple monitoring and full-field strain measurement, as well as prospective clinical studies integrating preoperative simulation, postoperative imaging, and longitudinal outcomes.

## 5 Conclusion

Stratification by simulated osteolytic defect-volume fraction (T) suggested distinct biomechanical patterns between ^125^I brachytherapy alone and ^125^I combined with PVP in this FEA. When T ≤ 30%, ^125^I brachytherapy alone was associated with limited changes in global vertebral stability, and the addition of PVP mainly increased local stiffness with concomitant stress elevation, without a consistent overall mechanical advantage. With larger simulated osteolytic defect volumes (T > 30%), ^125^I combined with PVP tended to provide more favorable stress redistribution and improved displacement control than ^125^I brachytherapy alone, with a more evident reconstructive benefit at T ≥ 50%. These defect-volume thresholds reflect model-derived trends rather than clinical cut-offs; therefore, the absolute values should be interpreted cautiously and may benefit from validation and calibration in larger, heterogeneous cohorts. In practice, the findings may serve as supportive information for MDT decision-making when considered alongside SINS and other clinical factors.

## Data Availability

The raw data supporting the conclusions of this article will be made available by the authors, without undue reservation.
