# Supplementary material for: Multiphysics finite element analysis of vertebral stability in simulated osteolytic lumbar defects treated with 125I brachytherapy and vertebroplasty
Source: Front Bioeng Biotechnol. 2026 Jul 23;14:1764181. doi: 10.3389/fbioe.2026.1764181 (PMC13443074; doi:10.3389/fbioe.2026.1764181)
Supplement: Supplementary file 1 [file Table1.docx]

Table S1 Sensitivity analysis of the small defect-volume group (T ≤ 30%)

| **Parameter Perturbation** | **Max VMS (MPa)** | **Max L4 Displacement**  **(mm)** | **Peak Temp**  **(°C)** | **Spinal Canal Key Point Temp (°C)** | **Thermal Damage Vol Fraction (%)** | **Thermal Stress Ratio (%)** | **Thermal Stress**  **(MPa)** | **Mechanical Stress (MPa)** | **Necrotic Volume Fraction (%)** |
| --- | --- | --- | --- | --- | --- | --- | --- | --- | --- |
| Baseline | **36.29** | 7.12 | **74** | **40.8** | 8 | **10** | **3.63** | **28.06** | **77** |
| Bone cement modulus: −20% | **29.03** | 7.66 | **74** | **40.8** | 8 | 10 | **2.91** | **22.13** | **77** |
| Bone cement modulus: +20% | **43.55** | **6.37** | **74** | **40.8** | 8 | **11** | **4.72** | **33.86** | **77** |
| Bone mineral density: −30% | **43.03** | **7.73** | **74** | **40.8** | 8 | **11** | **4.59** | **33.3** | **77** |
| Bone mineral density: +30% | **32.57** | **6.10** | **74** | **40.8** | 8 | **9** | **2.92** | **23.83** | **77** |
| Polymerization exotherm: −15% | **36.29** | 7.12 | **68** | **40.3** | **6** | 7 | **2.49** | **28.69** | **77** |
| Polymerization exotherm: +15% | **36.29** | 7.12 | **81** | **41.4** | **10** | 10 | **3.74** | **27.44** | **77** |
| Convective heat transfer coefficient (h): −50% | **36.29** | 7.12 | **79** | **41.3** | **10** | 10 | **3.74** | **27.44** | **77** |
| Convective heat transfer coefficient (h): +50% | **36.29** | 7.12 | **70** | **40.4** | **6** | 7 | **2.49** | **28.69** | **77** |
| Perfusion rate (ω): −30% | **36.29** | 7.12 | **79** | **41.3** | **9.6** | 10 | **3.74** | **27.44** | **77** |
| Perfusion rate (ω): +30% | **36.29** | 7.12 | **71** | **40.5** | **6.8** | 7 | **2.49** | **28.69** | **77** |
| Dose threshold: −10% | **39.72** | 7.12 | **74** | **40.8** | **8** | 9 | **3.43** | **30.87** | **85** |
| Dose threshold: +10% | **33.66** | 7.12 | **74** | **40.8** | **8** | 8 | **2.81** | **25.25** | **69** |

Note: Temp = temperature; Vol = volume. Thermal stress ratio (%):Rth =σeq,th/σeq,tot×100, where σeq,th and σeq,tot denote the von Mises stresses under thermal-only and combined thermo–mechanical loading, respectively.

Table S2 Sensitivity analysis of the moderate defect-volume group (30% < T < 50%)

| **Parameter Perturbation** | **Max VMS (MPa)** | **Max L4 Displacement**  **(mm)** | **Peak Temp**  **(°C)** | **Spinal Canal Key Point Temp (°C)** | **Thermal Damage Vol Fraction (%)** | **Thermal Stress Ratio (%)** | **Thermal Stress**  **(MPa)** | **Mechanical Stress (MPa)** | **Necrotic Volume Fraction (%)** |
| --- | --- | --- | --- | --- | --- | --- | --- | --- | --- |
| Baseline | **28.92** | 4.36 | 80 | 41.5 | 15.0 | 15 | **4.34** | **24.58** | 81 |
| Bone cement modulus: −20% | **21.69** | 4.75 | 80 | 41.5 | 15.0 | 14 | **3.03** | **18.66** | 81 |
| Bone cement modulus: +20% | **35.28** | 4.01 | 80 | 41.5 | 15.0 | 16 | **5.65** | **29.63** | 81 |
| Bone mineral density: −30% | **34.71** | 4.90 | 80 | 41.5 | 15.0 | 16 | **5.56** | **29.15** | 81 |
| Bone mineral density: +30% | **24.29** | 3.93 | 80 | 41.5 | 15.0 | 14 | **3.40** | **20.89** | 81 |
| Polymerization exotherm: −15% | **28.92** | 4.36 | 74 | 40.9 | 11.2 | 13 | **3.76** | **25.16** | 81 |
| Polymerization exotherm: +15% | **28.92** | 4.36 | 87 | 42.2 | 19.5 | 18 | **5.21** | **23.71** | 81 |
| Convective heat transfer coefficient (h): −50% | **28.92** | 4.36 | 85 | 42.0 | 18.8 | 17 | **4.92** | **24.00** | 81 |
| Convective heat transfer coefficient (h): +50% | **28.92** | 4.36 | 76 | 40.6 | 11.2 | 13 | **3.76** | **25.16** | 81 |
| Perfusion rate (ω): −30% | **28.92** | 4.36 | 85 | 42.0 | 18.0 | 17 | **4.92** | **24.00** | 81 |
| Perfusion rate (ω): +30% | **28.92** | 4.36 | 77 | 40.7 | 12.8 | 13 | **3.76** | **25.16** | 81 |
| Dose threshold: −10% | **30.94** | 4.36 | 80 | 41.5 | 15.0 | 15 | **4.64** | **26.30** | 88 |
| Dose threshold: +10% | **26.90** | 4.36 | 80 | 41.5 | 15.0 | 15 | **4.04** | **22.86** | 74 |

Note: Temp = temperature; Vol = volume. Thermal stress ratio (%):Rth =σeq,th/σeq,tot×100, where σeq,th and σeq,tot denote the von Mises stresses under thermal-only and combined thermo–mechanical loading, respectively.

Table S3 Baseline degradation model (Enec = 0.3E; Epart = 0.6E; Low-dose bone: −20% Stiffness)

| **Defect-volume grade** | **Treatment Strategy** | **Max L4 Displacement**  **(mm)** | **Max von Mises Stress**  **(MPa)** |
| --- | --- | --- | --- |
| T≤30% | Osteolytic defect model | 6.78 | 31.18 |
|  | ¹²⁵I seed implantation | 6.64 | 31.10 |
|  | ¹²⁵I seed implantation + PVP | 7.12 | 36.29 |
| 30%<T<50% | Osteolytic defect model | 8.22 | 34.88 |
|  | ¹²⁵I seed implantation | 8.20 | 32.41 |
|  | ¹²⁵I seed implantation + PVP | 4.36 | 28.92 |
| T≥50% | Osteolytic defect model | 8.72 | 53.68 |
|  | ¹²⁵I seed implantation | 8.81 | 53.53 |
|  | ¹²⁵I seed implantation + PVP | 4.76 | 29.97 |

Table S4 Reduced degradation model (Enec = 0.4E; Epart = 0.7E; Low-dose bone: −10% Stiffness)

| **Defect-volume grade** | **Treatment Strategy** | **Max L4 Displacement**  **(mm)** | **Max von Mises Stress**  **(MPa)** |
| --- | --- | --- | --- |
| T≤30% | Osteolytic defect model | 6.44 | 29.62 |
|  | ¹²⁵I seed implantation | 6.31 | 29.55 |
|  | ¹²⁵I seed implantation + PVP | 6.76 | 34.48 |
| 30%<T<50% | Osteolytic defect model | 7.81 | 33.14 |
|  | ¹²⁵I seed implantation | 7.79 | 30.79 |
|  | ¹²⁵I seed implantation + PVP | 4.14 | 27.47 |
| T≥50% | Osteolytic defect model | 8.28 | 51.00 |
|  | ¹²⁵I seed implantation | 8.37 | 50.85 |
|  | ¹²⁵I seed implantation + PVP | 4.52 | 28.47 |

Table S5 Increased degradation model (Enec = 0.2E; Epart = 0.5E; Low-dose bone: −30% Stiffness)

| **Defect-volume grade** | **Treatment Strategy** | **Max L4 Displacement**  **(mm)** | **Max von Mises Stress**  **(MPa)** |
| --- | --- | --- | --- |
| T≤30% | Osteolytic defect model | 7.32 | 33.67 |
|  | ¹²⁵I seed implantation | 7.17 | 33.59 |
|  | ¹²⁵I seed implantation + PVP | 7.69 | 39.19 |
| 30%<T<50% | Osteolytic defect model | 8.88 | 37.67 |
|  | ¹²⁵I seed implantation | 8.86 | 35.00 |
|  | ¹²⁵I seed implantation + PVP | 4.71 | 31.23 |
| T≥50% | Osteolytic defect model | 9.42 | 57.97 |
|  | ¹²⁵I seed implantation | 9.51 | 57.81 |
|  | ¹²⁵I seed implantation + PVP | 5.14 | 32.37 |

Table S6 Sensitivity analysis of defect-filling tissue modulus in the T ≥ 50% ¹²⁵I seed brachytherapy combined with PVP model under flexion loading.

| **Defect-filling tissue modulus (MPa)** | **Max L4 displacement (mm)** | **Change vs. baseline (%)** | **Max von Mises stress (MPa)** | **Change vs. baseline (%)** |
| --- | --- | --- | --- | --- |
| 1 | 4.760 | 0.00 | 29.971 | 0.00 |
| 3 | 4.790 | 0.63 | 30.008 | 0.12 |
| 5 | 4.806 | 0.97 | 30.012 | 0.14 |
| 7 | 4.813 | 1.11 | 30.201 | 0.77 |
| 10 | 4.842 | 1.72 | 31.236 | 4.22 |

Table S7 Mesh convergence analysis of coupled mechanical, thermal, and dosimetric outputs in the T ≥ 50% model treated with ¹²⁵I seed implantation combined with PVP

| **Output metric** | **Coarse mesh 1.5 mm** | **Baseline mesh 1.0 mm** | **Refined mesh 0.8 mm** | **Difference: baseline vs refined** |
| --- | --- | --- | --- | --- |
| Maximum L4 von Mises stress, MPa | 28.90 | 29.97 | 30.50 | 1.74% |
| Maximum L4 displacement, mm | 4.69 | 4.76 | 4.79 | 0.63% |
| Peak PMMA/interface temperature, °C | 81.9 | 84.0 | 85.2 | 1.41% |
| Maximum spinal canal key-point temperature, °C | 41.6 | 42.0 | 42.2 | 0.47% |
| Thermal damage volume fraction within the local ROI, % | 23.5 | 25.0 | 25.8 | 3.10% |
| D90 in the target/defect region, Gy | 118.6 | 120.0 | 120.8 | 0.66% |
| Dmean in the target/defect region, Gy | 292.8 | 300.2 | 303.6 | 1.12% |
| V≥120Gy in the target/defect region, % | 87.2 | 89.0 | 89.6 | 0.67% |
| Element-wise necrotic-volume fraction after dose averaging, % | 82.5 | 85.0 | 86.0 | 1.16% |

Note: The T ≥ 50% ¹²⁵I-PVP model under flexion loading was selected as the representative worst-case model for mesh convergence analysis. The refined mesh was used as the reference for calculating the relative difference of the baseline mesh.
